# Approaching the Distinction between Intuition and Insight

**DOI:** 10.3389/fpsyg.2016.01195

**Published:** 2016-08-09

**Authors:** Zhonglu Zhang, Yi Lei, Hong Li

**Affiliations:** Research Centre for Brain Function and Psychological Science, Shenzhen UniversityShenzhen, China

**Keywords:** intuition, insight, judgment, solution, RAT, tacit knowledge

## Abstract

Intuition and insight share similar cognitive and neural basis. Though, there are still some essential differences between the two. Here in this short review, we discriminated between intuition, and insight in two aspects. First, intuition, and insight are toward different aspects of information processing. Whereas intuition involves judgment about “yes or no,” insight is related to “what” is the solution. Second, tacit knowledge play different roles in between intuition and insight. On the one hand, tacit knowledge is conducive to intuitive judgment. On the other hand, tacit knowledge may first impede but later facilitate insight occurrence. Furthermore, we share theoretical, and methodological views on how to access the distinction between intuition and insight.

## Background

Intuition can be conceived of as a sudden apprehension of coherence (pattern, meaning, structure) above chance level with little conscious retrieval (Bowers et al., 1990; Bolte et al., 2003; Bolte and Goschke, 2005; Volz and Von Cramon, 2006; Ilg et al., 2007; Topolinski and Strack, 2008; Topolinski, 2011). By contrast, insight is defined as a sudden access to solution by restructuring, or changing problem representation (Ohlsson, 1984; Knoblich et al., 1999; Öllinger and Knoblich, 2009; Öllinger et al., 2013; Kounios and Beeman, 2014). The nature of intuition or insight has been empirically investigated and theoretically discussed in literature, separately. However, quite few theoretical discussions address the relationships between the two. In fact, they share many commons and are intimately linked with each other. For example, both occur under somewhat similar situations where the final results are not clear. That is, an intuitive judgment would be made under an uncertain circumstance perhaps due to time pressure or lack of sources (Kahneman, 2003) or for insight an impasse would be encountered beforehand where individuals do not know what to do next though they have made great efforts (Ohlsson, 1984; Knoblich et al., 1999). In addition, both intuition, and insight rely on the unconscious spreading activation of semantic associates (Ohlsson, 1984; Bowers et al., 1990; Bowden and Beeman, 1998; Jung-Beeman et al., 2004; Bolte and Goschke, 2005; Ilg et al., 2007; Cai et al., 2009; Sio et al., 2013) and the activation of the right superior temporal cortex (Jung-Beeman et al., 2004; Ilg et al., 2007). In line with this, they share a common counterpart for comparison, namely the analytic process which operates in a deliberately controlled style under the framework of the dual-process theory (Epstein, 1994; Sloman, 1996; Stanovich and West, 2000; Kahneman, 2003). Moreover, fluency, as the relative speed and efficiency of information processing (Reber et al., 2004), plays a causal role in both phenomena. Processing fluency of the encoded material (without actually retrieving the solution) is the driving force of the gut feeling of intuition not only in the coherence judgment (e.g., Topolinski and Strack, 2009; Topolinski, 2011) but also in the intuitive judgment for solvability of problems (e.g., Topolinski et al., 2016) and in insight the fluency of solution retrieval is a rather epiphenomenal factor that does not cause the insight itself, but that elicits its distinctive experiential feature (“Aha” feeling) (Topolinski and Reber, 2010).

## Differences between intuition and insight

Though intuition and insight share overlapping cognitive and neural features, as summarized above, they are actually not the same coin, and can be essentially differentiated from each other to large extent. Some works have addressed the differences between them. For example, insight comes after intuition, and appears into consciousness (Volz and Von Cramon, 2006). In addition, intuition is continuous whereas insight is discontinuous (e.g., Bowers et al., 1990). Furthermore, as Reber et al. (2007) showed, there are significant increase in both subjective closeness and objective closeness in intuitive judgment whereas subjective closeness is not significantly increased, lagging far behind objective closeness in insight problem solving. Obviously, the behavioral, and phenomenological differences have been well documented. Moreover, we propose that intuition and insight are different from each other not only in the behavioral and phenomenological levels but also in the cognitive levels in essence. We will discuss them as follows in two aspects.

First, intuition and insight are toward two distinctive aspects of information processing. Though the unconsciously activated information plays a common and fundamental role in both intuition (e.g., Bolte and Goschke, 2005; Ilg et al., 2007) and insight (e.g., Jung-Beeman et al., 2004; Sio et al., 2013), it is guided by different cognitive operations. For intuition, this unconsciously activated information is guided by an intuitive judgment task on whether there is a coherence or a fourth associative word for the triads. More specifically, intuition mainly involves the processing of judgment on “yes/no, ” namely intuitive judgment, which is intimately related to the behavior of decision making (Tversky and Kahneman, 1974; Dane and Pratt, 2007, 2009). In this regard, intuition cares little about “what the ultimate result is” but the individuals' subjective decision upon whether there is a solution or not. For insight, however, this unconsciously activated information is guided by conscious retrieval which requires accessing the insightful solutions (the fourth associative word for the triads). In other words, insight is something about “what” is the solution rather than judgment. Evidences from the functional magnetic resonance imaging (fMRI) studies support the views above to some extent. With the Remote Associate Test (RAT; Mednick and Mednick, 1967), Ilg et al. (2007) and Jung-Beeman et al. (2004) investigated the neural basis of intuition and insight, respectively. Both found activities in the right superior temporal cortex, which was regarded to be reflecting the common role of the unconsciously activated information (Ilg et al., 2007). Moreover, they found extra neural activity that can distinguish different cognitive operations (intuitive judgment vs. retrieving insightful solutions) on the unconsciously activated information. Specifically, the task of intuitive judgment activates brain areas such as the bilateral inferior parietal cortex that are generally related to the process of decision making under uncertainty (Paulus et al., 2001; Ilg et al., 2007). On the other hand, the task of retrieving insightful solutions elicited a gamma-band activity, which indexes the accessibility into conscious representations (Engel and Singer, 2001; Jung-Beeman et al., 2004).

Second, the role of tacit knowledge in intuition and insight should be different. Intuition mainly benefits from tacit knowledge. Activation of tacit knowledge starts to spread from the three concepts (e.g., in the RAT) and finally converges on the common remote associate. As activation accumulates, it can facilitate the intuitive judgment though not trigger conscious retrieval (Ilg et al., 2007). Meanwhile, this accumulated activation brings individuals the feeling of subjective closeness to the solution (Reber et al., 2007). The whole processing stream starting from the primary activation of tacit knowledge to final intuitive judgment goes continuously instead of discontinuously without any barrier (Bowers et al., 1990). All these indicate that tacit knowledge benefits the processing of intuitive judgment of coherence, resulting in a continuous pattern. In contrast, tacit knowledge may play double roles (first harmful and then helpful) in insight occurrence. In this sense, tacit knowledge can be divided into valid and invalid categories. In insight problem solving, solvers primarily encounter impasse, which is mainly caused by the strong activations of unhelpful tacit knowledge (Ohlsson, 1984; Knoblich et al., 1999, 2001). The impasse can be overcome when weak but valid tacit knowledge can be activated and accessed (Knoblich et al., 2001; Bowden and Jung-Beeman, 2007) and this mainly relies on the activities at the right anterior superior temporal gyrus (Jung-Beeman et al., 2004; Bowden and Jung-Beeman, 2007).

## Approaching the distinction between intuition and insight: theoretical and methodological proposals

As aforementioned, intuition and insight are two mutually related but different cognitive constructs. However, the differences (as well as the commonalities) that summarized above are just based on the theoretical and empirical data in the respective field of intuition and insight. To better understand the nature of intuition and insight, two concerns should be taken into consideration. First, to what extent intuition and insight are related and distinguished with each other? Second, there is lack of research that can systematically and directly examine their mechanisms in the same experiment thus far. In this vein, we share our viewpoints below.

Theoretically, future researches can consider how the unconsciously activated information interacts with intuitive judgment and the conscious retrieval of insightful solutions, respectively. Though there have been some neuroimaging evidences, as we summarized that can partly support the view that the unconsciously activated information is guided by different cognitive operations (namely “yes/no” judgment for intuition and conscious retrieval of solutions for insight, respectively), relevant studies in both fields are relatively few and need to be further replicated, and expanded. In addition, as we have distinguished, tacit knowledge may play different role in between intuition, and insight. Some tacit knowledge may be helpful for intuitive judgment but harmful for insight occurrence (and vice versa). We suggest that more empirical studies can be conducted to examine how tacit knowledge influence intuition and insight.

In methodology, we propose that future researches can directly examine, and compare the cognitive and neural mechanisms between intuition and insight in the same experiment and this is possible for two reasons. First, the commonly used materials—the RAT—have been widely used in the studies of both intuition (e.g., Bowers et al., 1990; Bolte et al., 2003; Bolte and Goschke, 2005; Ilg et al., 2007; Topolinski and Strack, 2008, 2009; Topolinski, 2011) and insight (e.g., Bowden and Beeman, 1998; Jung-Beeman et al., 2004; Cai et al., 2009; Sio et al., 2013). The RAT consist of a certain number of items and in each item there are three words of a triad as well as their common associate (the solution word; Mednick, 1962; Mednick and Mednick, 1967). For example, the triad “night, wrist, stop” are in association with the solution word “watch.” In insight problem solving, the task for the participants is to retrieve the solution word according to the three words. Only those solutions accompanied by “aha” feelings are regarded as insightful ones (e.g., Bowden and Jung-Beeman, 2003, 2007; Jung-Beeman et al., 2004). In intuitive judgment task, there are not only the coherent triads (e.g., “night, wrist, stop”) with their common associates but also the incoherent triads (e.g., “house, lion, butter”) without any common associate. Participants do not need to retrieve the solution word but judge whether the triads are coherent or not (e.g., Bolte and Goschke, 2005; Ilg et al., 2007). Second, intuition and insight stay at different phases in the stream of information processing. Intuition occurs at the moment of coherence judgment with the potential solutions not retrieved (Ilg et al., 2007). Insight, however, comes at a later stage (Volz and Von Cramon, 2006), occurring at the moment of solution retrieval (Jung-Beeman et al., 2004) which cannot be predicted by the intuitive judgment of FOK (feeling of knowing) (Metcalfe and Wiebe, 1987). Considering these two points, we suggest that they can be measured subsequently in one experimental paradigm with the RAT as the materials. A general paradigm is developed as follows (it should be noted that this is one but not the only way to explore the differences between intuition and insight).

**Figure 1:**
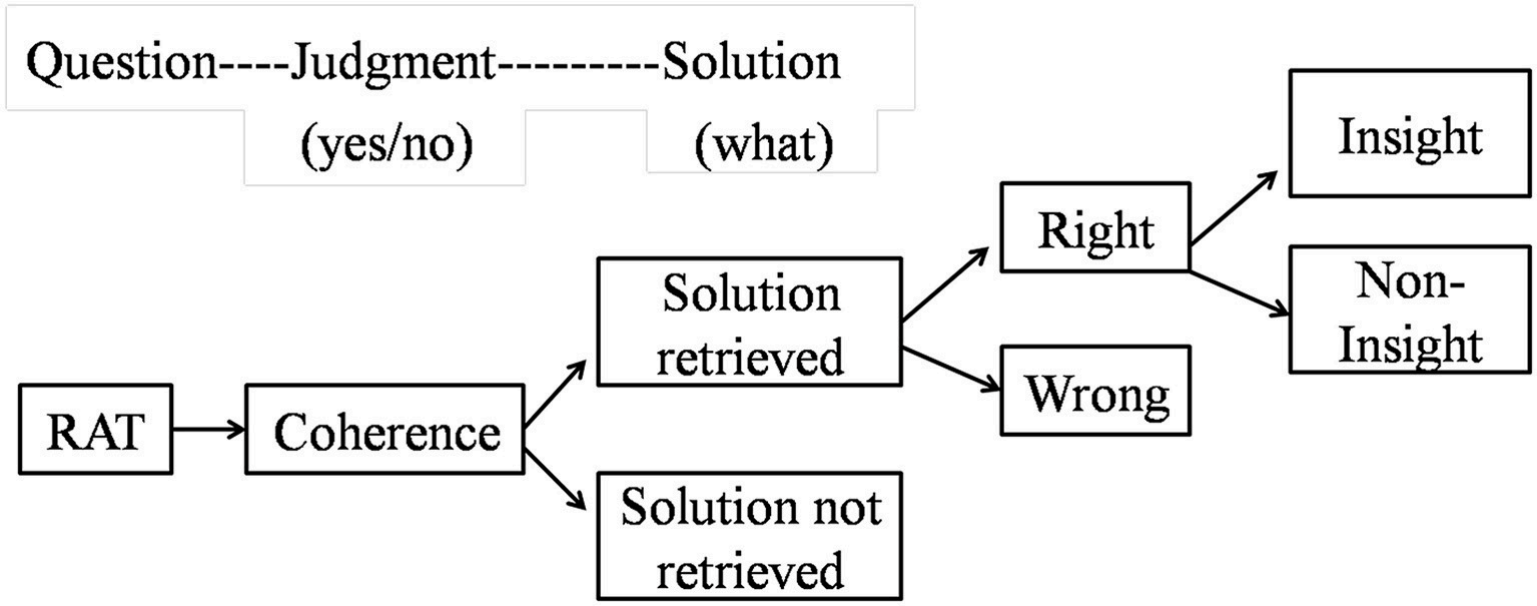
**The experimental procedure for both intuitive judgments and insightful solutions**. In the question phase, the word triads are presented; In the judgment phase, participants are asked to judge whether the word triads are coherent or not; In the solution phase, participant are asked to retrieve the solutions. Intuition can be measured at the moment of coherent judgment with the solution not retrieved. Insight can be measured when the right solutions are retrieved and reported to be insightful.

As described in Figure 1, the RAT (the coherent triads with solutions) as well as the incoherent triads (without solutions) can be congregated together and then be randomly presented to the participants one by one. Considering that the intuitive judgment and the solutions retrieval stay at different phases in the stream of information processing in problem solving, participants can be instructed to complete the two tasks subsequently. Specifically, participants can receive the coherence judgment task first, in which they are asked to judge whether the word triads have a common associate. In light of previous researches (e.g., Bolte et al., 2003; Bolte and Goschke, 2005; Ilg et al., 2007), intuition can be measured when the coherence judgments were made with the solutions not retrieved. After the coherence judgment task, participants can be told to retrieve the solutions to the problems. According to previous literature (Jung-Beeman et al., 2004; Bowden and Jung-Beeman, 2007), insight can be measured at the moment of correct solutions retrieved which are reported insightful.

Furthermore, researchers can investigate and compare the cognitive and neural basis of intuition and insight based on the above-introduced paradigm by utilizing the brain imaging techniques such as fMRI, electroencephalograph (EEG), and so on. For example, with high spatial resolution, fMRI can be used to localize “where” the neural signals related to the cognitive events are in the level of millimeter in space. fMRI has been used in the fields of both intuition and insight and the relevant studies have found some brain region such as the right superior temporal cortex activated in intuition and insight (Jung-Beeman et al., 2004; Ilg et al., 2007). This provides potential regions of interest (ROI), based on which future researches can build their respective hypothesis and further examine the neural basis of intuition and insight. Similarly, with millisecond-level temporal resolution, EEG would be useful in elucidating the neural correlates of intuition, or insight by providing neural marks such as the event-related potentials (e.g., N100, N200, P300) in time domain or the neural oscillations (e.g., alpha, beta, gamma) in frequency domain. With RAT test, Jung-Beeman et al. (2004) found a gamma-band oscillation associated with conscious retrieval in insight problem solving. In addition, they observed an alpha burst preceding the gamma burst. This insight-specific alpha effect may reflect unconscious solution-related processing (Jung-Beeman et al., 2004). By contrast, there are few EEG studies of intuition. Thus, one straightforward hypothesis would be, for example, could alpha-band oscillation, or gamma-band oscillation be observed during the moment of intuition? In short, the brain imaging techniques would help to prosper the fields of both intuition and insight.

## Conclusions

As we summarized, intuition, and insight can be essentially differentiated from each other when considering whether the unconsciously activated information is guided by intuitive judgment or conscious retrieval and the different roles of tacit knowledge. Nevertheless, the differences may not be just limited to these two aspects, which in fact need more empirical examinations and evidences. We propose that by means of the brain imaging techniques, future researches can consider directly examining the cognitive and neural mechanisms of both intuition and insight based on the RAT in one experiment.

## Author contributions

ZZ drafted the manuscript, YL and HL provided critical revisions.

### Conflict of interest statement

The authors declare that the research was conducted in the absence of any commercial or financial relationships that could be construed as a potential conflict of interest.
